# Doping Control Using High and Ultra-High Resolution Mass Spectrometry Based Non-Targeted Metabolomics-A Case Study of Salbutamol and Budesonide Abuse

**DOI:** 10.1371/journal.pone.0074584

**Published:** 2013-09-18

**Authors:** Agneta Kiss, Marianna Lucio, Aurélie Fildier, Corinne Buisson, Philippe Schmitt-Kopplin, Cécile Cren-Olivé

**Affiliations:** 1 EquipeTRACES, Institut des Sciences Analytiques-UMR, Villeurbanne, France; 2 Helmholtz Zentrum München, German Research Center for Environmental Health, Neuherberg, Germany; 3 Département des analyses, Agence Française de Lutte contre le Dopage (AFLD), Châtenay-Malabry, France; 4 Chair of Analytical Food Chemistry, Technische Universität München, Freising-Weihenstephan, Germany; University of Jaén, Spain

## Abstract

We have detected differences in metabolite levels between doped athletes, clean athletes, and volunteers (non athletes). This outcome is obtained by comparing results of measurements from two analytical platforms: UHPLC-QTOF/MS and FT-ICR/MS. Twenty-seven urine samples tested positive for glucocorticoids or beta-2-agonists and twenty samples coming from volunteers and clean athletes were analyzed with the two different mass spectrometry approaches using both positive and negative electrospray ionization modes. Urine is a highly complex matrix containing thousands of metabolites having different chemical properties and a high dynamic range. We used multivariate analysis techniques to unravel this huge data set. Thus, the several groups we created were studied by Principal Components Analysis (PCA) and Partial Least Square regression (PLS-DA and OPLS) in the search of discriminating m/z values. The selected variables were annotated and placed on pathway by using MassTRIX.

## Introduction

The molecular diversity of the human urinary metabolome is very well reflected by the existing databases [1,2] displaying thousands of metabolites classified in as much as 70 different structural classes [3-5]. The majority of these metabolites are small molecular weight compounds having molecular weights between 100 and 800 Dalton (Da) and small peptide fragments [6] with very different physico-chemical properties (solubility, polarity, proton affinity, etc). Despite this astonishing chemo-diversity, for quite some time, mainly targeted studies, often restricted to a particular chemical family or to compounds having similar properties have been applied into doping control studies. Moreover, the metabolites and the changes were often regarded in an univariate manner and the correlations between them were often disregarded.

Nowadays, the progresses made in analytical fields like sample preparation, chemical analysis and data processing offer a wider view of the metabolome and greatly contribute to our understanding of the biochemical transformations. Among these, two are believed to have played a key role: the introduction of high (Time of Flight mass spectrometry, TOF) and ultra-high resolution techniques (Fourier Transform Ion Cyclotron Resonance mass spectrometry, FT-ICR/MS) and the development of algorithms capable of handling the thousands of signals generated by such analytical platforms. Indeed, techniques such as FT-ICR/MS are becoming more and more available and their advantages can be now fully exploited. Thus, molecular formulae generation based on exact masses and relevant database search are now possible due to the high resolution and accuracy of this type of technique [7,8]. Of equal importance, the recent advances in the pre-processing [9], mathematical modeling [10,11] and the statistical analysis [10] lead to more comprehensive biological interpretation of the metabolomics data [12]. Up to present, this strategy has been applied in a variety of fields, including drug discovery [13,14], nutrition [15,16], toxicology [17], clinical trials [18] and more recently chemical submission [19]. It is of particular interest in areas like doping control as new approaches are needed to fight the ongoing development of performance-enhancing methods [20-23].

In this study, this strategy was applied to real urine samples collected from volunteers (V), high-level clean athletes (CA) and athletes declared positive for the use of beta-2-agonists (salbutamol, S) or glucocorticoids (budesonide, B). The choice of the doping agents was based on three criteria: the high frequency detection in sport [24], the common medical use in asthma treatment and respiratory conditions [25] and the excretion through urine [26,27]. The main purposes of this paper are to explore (i) the metabolic differences between volunteers and sportsmen and (ii) the metabolic changes induced by the intake of budesonide and salbutamol.

## Materials and Methods

### Study design and general strategy

**Figure 1:**
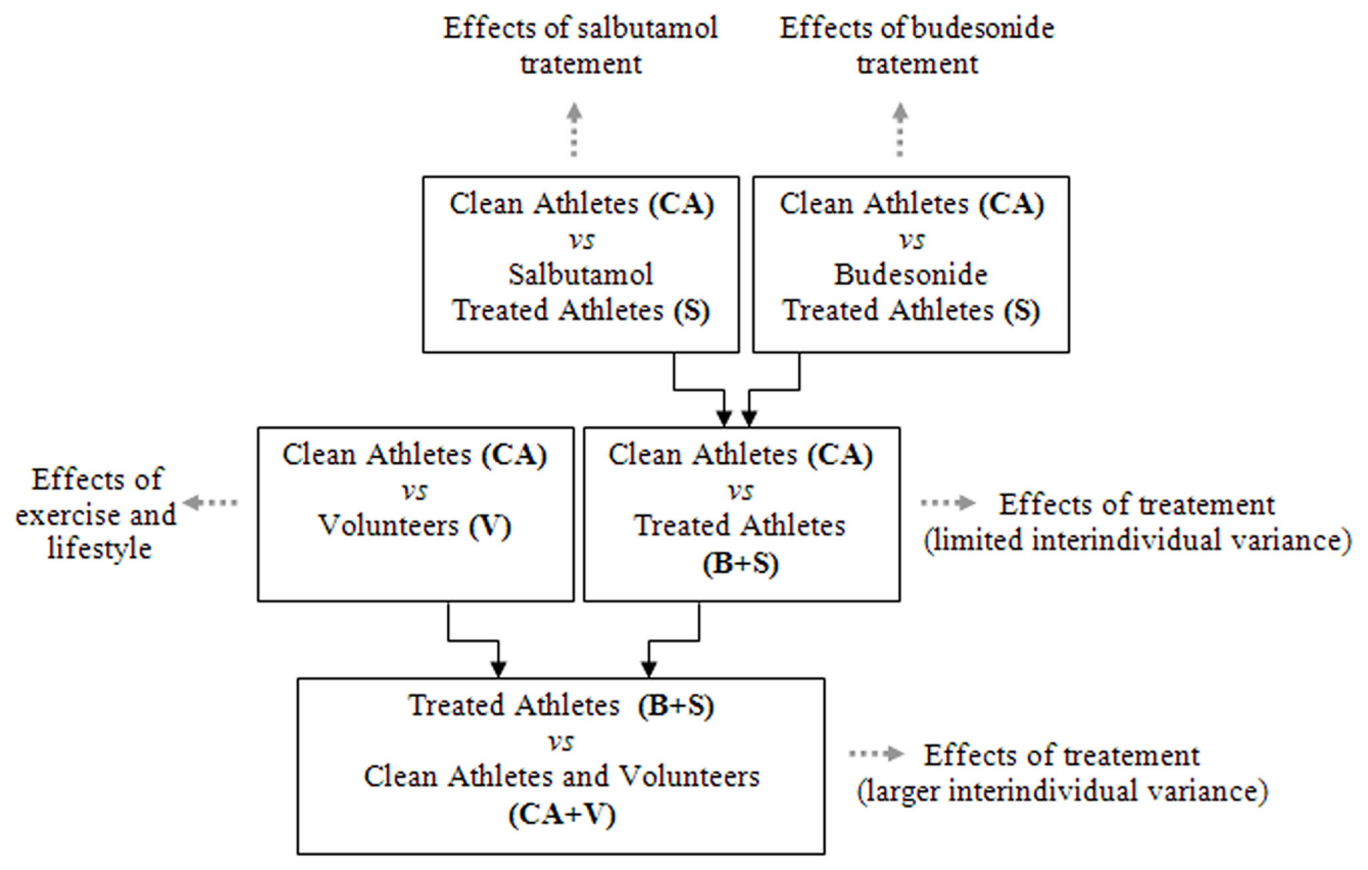
Study design and general strategy.

Firstly, the spectra acquired with FT-ICR/MS were preprocessed and prepared for analysis. Several groups (Figure 1) were selected and treated independently via mathematical and statistical methods such as Principal Component Analysis (PCA, unsupervised) and Orthogonal Partial Least Square (OPLS, supervised). The two models involving budesonide and salbutamol treated athletes were then compared in order to emphasize the metabolic similarities and differences. Finally, we compare the data obtained by direct injection into the FT-ICR mass spectrometer with the one obtained by including a separation step in the analysis framework. Thus, the LC-QTOF data set was compared with the FT-ICR/MS data set by means of PCA-CCA (Principal Components Analysis-Canonical Correlation Analysis).

### Sample Collection

The protocol was approved by the Institutional Review Board of the University of Lyon, F-69622, forty-three bd du 11 novembre, Villeurbanne, and the Institutional Review Board of the French Anti-Doping Agency, 143, avenue Roger Salengro, Châtenay-Malabry (according to the Declaration of Helsinki). All subjects gave written informed consent before the study commenced. The investigation was conducted in accordance with the ethical principles of Good Clinical Practice. The experiment includes 11 urine samples coming from clean athletes, 9 urine samples collected from volunteers and 27 urine samples provided by the French Anti-Doping Agency (AFLD, Chatenay-Malabry) and coming from athletes declared positive for illicit substance consumption. Among the 27 positive samples, 17 samples belong to salbutamol doped athletes and 10 samples belong to budesonide doped athletes. The urine samples coming from volunteers were collected in comparable conditions and by following the AFLD guidelines. All urine samples were aliquoted and stored at -24°C until analysis, as recommended for stability reasons [27].

### Sample Preparation

A “quality control” (QC) sample was prepared as recommended in different publications [28-30] by mixing equal volumes (50 µL) of each of the samples as they were being aliquoted. This “pooled” urine was used to provide a representative *mean* sample containing all the analytes that will be encountered during the analysis and it was mainly used to assess the quality of the data. At the beginning of the batch, 3 methanol samples were run to ensure that the analytical system had come to equilibrium. The QC samples were injected once at the beginning, in the middle and at the end of the batch. For the FT-ICR analysis, all the samples were diluted 50 times in methanol and vortexed for 2 minutes at 10000 rpm. The supernatant was then taken and injected. For the UHPLC-QTOF analysis the urine samples were injected as neat.

### Experimental Conditions

#### Fourier Transform Ion Cyclotron (FT-ICR MS)

High-resolution mass spectra were acquired on a Bruker (Bremen, Germany) SolariX Fourier Transform Ion Cyclotron Resonance MS equipped with a 12 Tesla superconducting magnet and an Apollo II electrospray source. The high magnetic field FT-ICR instrument combines the highest resolution (400.000 at m/z 400) and the best mass accuracy (<200 ppb) and thus enables the direct conversion of the experimental mass into elementary composition. The urine samples were infused with the micro-electrospray source at a flow rate of 120 mL/h The The nebulizer gas pressure and the drying gas pressure were fixed at 20 psi and 15 psi, respectively. The source temperature was 250°C. Data was acquired in both positive and negative electrospray modes. The mass spectrometer was externally calibrated at the beginning of the batch. The calibration solution consisted of arginine clusters (m/z of 173.10440, 347.21607, 521.32775 and 695, 43943) dissolved in methanol at a concentration of 10 mg/L. Calibration errors in the relevant mass range were always below 0.1 ppm (part per million).

#### Liquid chromatography coupled to quadrupole time of flight mass spectrometry (UHPLC-QTOF-MS)

The UHPLC-MS platform consisted of a chromatographic system suitable for high resolution separations (Dionex Ultimate 3000) coupled with a quadrupole time-of-flight (QTOF) mass detector equipped with electrospray ionization (microTOF QII, Bruker Daltonics). The chromatographic separation was conducted on an Acquity C18 BEH column (100mm x 2.1 mm, 1.7 µm). The column oven temperature was set to 50°C, injection volume at 5 µL and flow-rate at 0.5 ml/min without a split. The eluents used were: (A) water acidified with 0.1% formic acid and (B) acetonitrile with 0.1% formic acid. The gradient was set as follows: 100% (A) over 2 min followed by a linear increase from 0 to 100% (B) over 30 min. An isocratic cleaning step at 100% (B) over 5 min and column equilibration step at 100% (A) over 15 min were used after each sample. Each urine sample was injected three times in order to assure reproducibility. Detection was performed in negative electrospray mode under the following conditions: nebulizer pressure 1 bar, dry gas flow 8 L/min, source temperature 120 °C, source temperature 200 °C, capillary voltage 3500 V. Mass spectrometric data were collected over the range 80-1000 m/z in profile mode at 1 spectra/second. A sodium formate solution was injected for 0.1 min at the beginning of each run for internal calibration.

### Data Analysis

The data analysis procedure followed in this paper consists of: internal calibration, peak identification, peak list alignment, mathematical and statistical analysis and database annotations.

The raw data (FT-ICR/MS and UHPLC-QTOF) were independently pre-processed with the proprietary software DataAnalysis (Bruker Daltonik, Bremen). The FT-ICR/MS acquired spectra were internally calibrated using a reference list: solvent impurities for the positive mode and fatty acids for negative mode in order to account for deviations occurring during the batch. Calibrations errors were found to be inferior to 0.2 ppm regardless of the ionization mode. Next, the peaks having a signal-to-noise ratio superior to four (4) were exported to individual peak lists. The peak lists were then aligned over the entire mass range through an in-house software with a tolerance of 1 ppm [31]. Profile Analysis (Bruker Daltonics) was used to internally calibrate and align the UHPLC-QTOF data in both m/z and retention time directions Tolerances of ±0.05 Da and ±0.2 min were used, respectively.

Prior to data analysis the FT-ICR/MS and UHPLC-QTOF data sets were Pareto-scaled. Modeling and statistical analysis were done with SIMCA-P+12 (Umetrics, Umeå, Sweden) and in MATLAB (v.10, Mathworks, USA). Several techniques were used in order to extract the information contained in the multivariate data sets. Firstly, Principal Component Analysis (PCA) was used in order to have a first overview of the FT-ICR/MS data and to detect general trends and relations between the spectra and the variables (m/z). Orthogonal Partial Least Squares regression (OPLS), was used in order to select those signals related to the nature of samples e.g. treated, clean, volunteer and athlete (supervised technique). As showed in Figure 1, the models were built for each comparison. These models were firstly validated by the 7 fold cross validation procedure implemented in the software and then with CV-ANOVA (Cross Validation ANOVA), in order to exclude the possible presence of over-fitting. Pertinence indicators such as: p-value, R^2^(Y) and Q^2^(cum), were subsequently reported for each model indicating the significance, the goodness of the fit and the goodness in the model prediction. Moreover potential discriminating variables were chosen by examining the S-plot (which combines the modeled covariance and correlation, variables with high value in magnitude and reliability can be candidate for the class separation) and VIP values (variable influence in the projection). Additionally graphical methods such as SUS-plot (Shared and Unique Structures) were used for model comparison. Canonical Correlation Analysis preceded by PCA was done in MATLAB in order to compare the output of the two platforms (UHPLC-QTOF and FT-ICR).

The masses characterizing the metabolic distinction were submitted to MassTRIX using *Homo sapiens* as reference species and a maximum error of 1 ppm. This web server assigns a potential annotation from KEGG (http://www.genome.jp/kegg/), LipidMaps (http://www.lipidmaps.org/) and HMDB [4,5] to each m/z value. As second step, MassTRIX calls the KEGG/API (http://www.genome.jp/kegg/soap/) to generate the pathway maps of the annotated masses [6].

## Results and Discussion

### FT-ICR/MS

**Figure 2:**
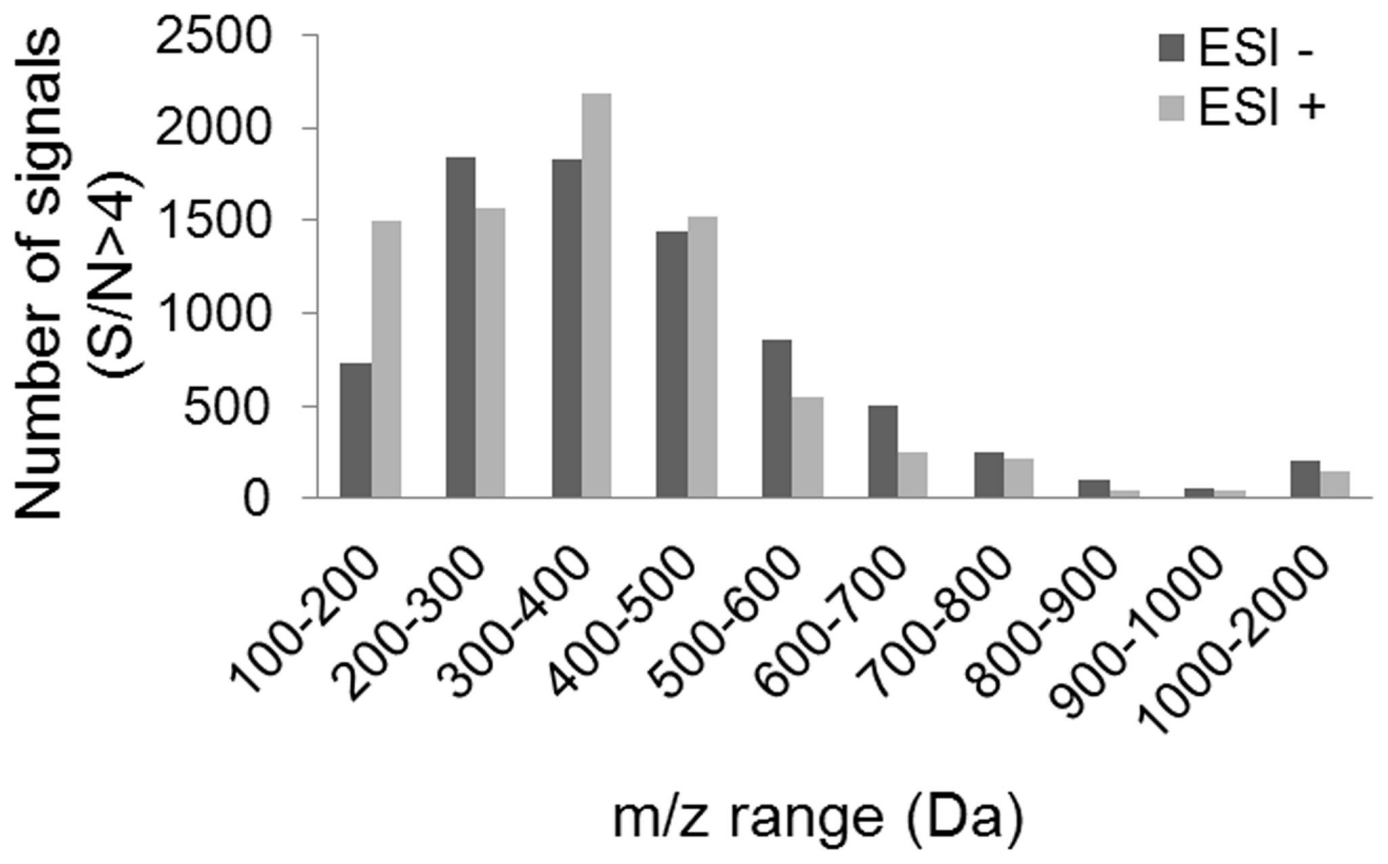
Distribution of the signals as a function of the mass range.

The alignment of the FT-ICR/MS peak lists lead two matrices corresponding to the two ionization modes. Initially the positive and the negative matrices counted 23.246 signals and 20.830 signals, respectively. The signals appearing in less than 5% of the samples were associated to noise and were excluded from further analysis. Thus, the positive and negative matrices underwent a serious downsizing to 63% and 89% of the initial number of signals, respectively. The majority of the remaining signals are situated between 150 and 500 Da and its distribution is shown in Figure 2.

### UHPLC-QTOF/MS

**Figure 3:**
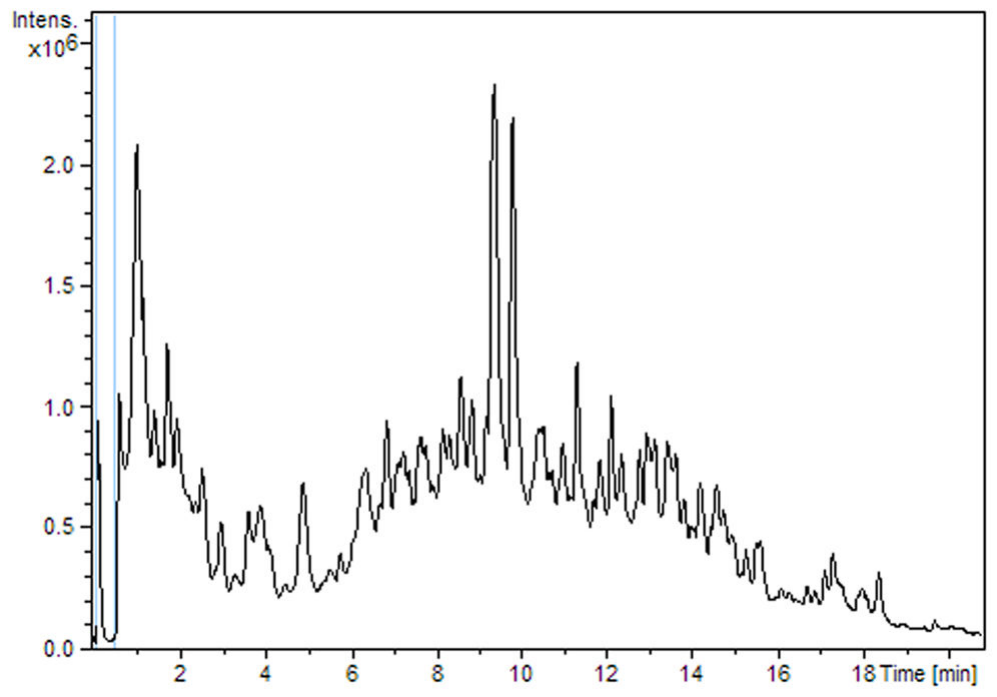
UHPLC-QTOF/MS chromatogram.

The positive mode (Figure 3) yielded 47133 variables. Some of these are metabolites while others are fragments, adducts, isotopes and noise. 40457 variables are present in less than five samples. As before, these variables were considered unreliable and were not included in the statistical analysis. 104 variables were detected in all the samples. The majority of the detected compounds have molecular weights between 100 and 700 Da. Since the distribution of the detected signals over the mass range is sensitive to the acquisition parameters of the mass spectrometer, a direct comparison of two distributions (UHPLC-QTOF and FT-ICR) would not be appropriate.

A detailed analysis of the data emphasises a wide range of polarity. Approximately 16% of the compounds are eluted before 20% acetonitrile and the vast majority of compounds are eluted before reaching 75% acetonitrile.

### Unsupervised Statistical Analysis

**Figure 4:**
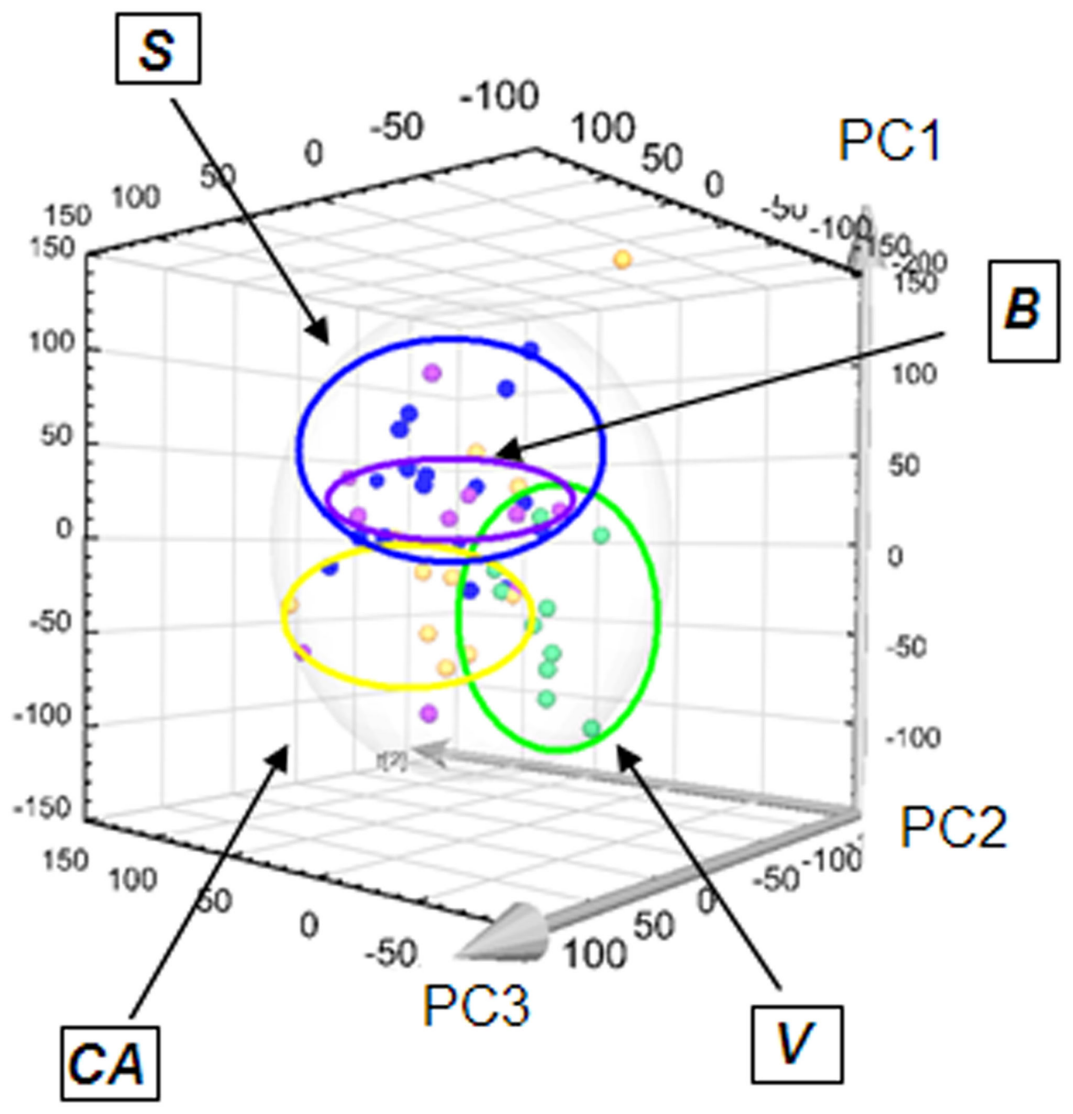
Principal Component Analysis showing clusters corresponding to the 4 groups (B, S, V, CA).

The PCA is used in order to get a first glance of the properties of the FT-ICR data. As a result of this analysis applied to the totality of the samples, a clustering of some samples becomes apparent by eye (see Figure 4). The 3D score plot shows that the (clean) volunteers (V) are more dispersed along the second principal component (Figure 4) suggesting a larger variability in the metabolism or the interference of different factors (diet, sport, medication, etc.).

The loadings plots are extremely complex and difficult to interpret due to the presence of many different groups. In order to enhance the differences between the groups and isolate the masses that discriminate them, supervised statistical methods were used. Models opposing the clean athletes and the volunteers (CA *vs* V), the clean athletes and the budesonide treated athletes (CA *vs* B) and the clean athletes and the salbutamol treated athletes (CA *vs* S) were created by using the OPLS statistical method. The values of significance for the FT-ICR models and UHPLC-QTOF models are reported in Table 1.

**Table 1:** Significance values for the OPLS models (CA vs B), (CA vs S) and (CA vs V).

	ESI+			ESI-		
	p	R2	Q2	p	R2	Q2
FT-ICR/MS models						
CA *vs* V	0.003	0.99	0.711	0.007	0.959	0.563
CA *vs* S	0.02	0.988	0.467	0.03	0.979	0.464
CA *vs* B	0.07	0.954	0.389	0.06	0.697	0.29
LC-QqToF models						
CA vs V	0.003	0.99	0.69	0.007	0.93	0.55
CA vs S	0.03	0.97	0.47	0.03	0.98	0.5
CA vs B	0.08	0.90	0.28	0.06	0.7	0.14

### (CA vs V): clean athletes vs volunteers

The aim of this study is to explore all the possible differences between the two groups from a global point of view and to emphasize the extent to which these differences interfere with metabolomics studies [32]. The differences between the metabolism of the non-athletes and that of high level athletes have already been the subject of many research papers. Thus, studies comparing the effects of endurance [33], sports type [34], effort degree and duration [35,36], can be found in literature. As the present study involves real urine samples, the emphasized contrast will originate not only in the physical effort, but also in any dietary, lifestyle or medical differences.

The OPLS model comparing the 10 volunteers to the 11 athletes controlled negatively by the AFLD, resulted in excellent R^2^(Y) (0.99), Q^2^(cum) (0.711) and p-values. (0.003). These values prove a strong relationship between the matrix of the measured variables (m/z signals) and the class membership in both positive and negative modes (Table 1).

**Figure 5:**
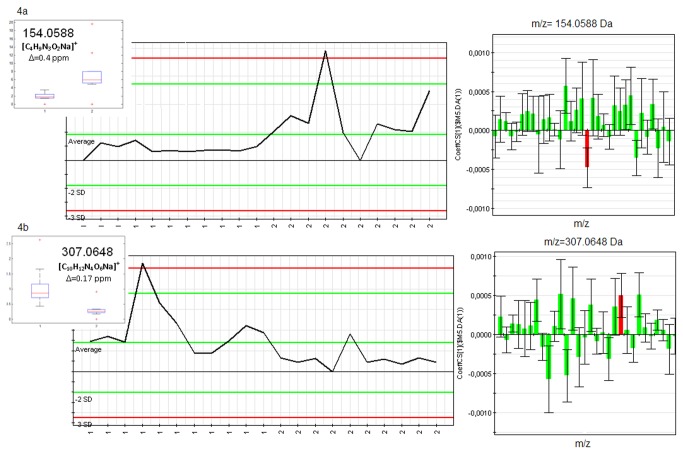
CA vs. V discriminant profiles: (a) creatine and (b) xanthosine.

Several discriminant signals were emphasized and selected based on the S-plot and the VIP values. An explanatory example of discriminant profiles having downtrend or uptrend behaviors with respect to CA are shown in Figure 5a (creatine) and Figure 5b (xanthosine), respectively. These signals were identified in the UHPLC-QTOF data under the form [M+H]^+^

Thus, for the positive mode of the FT-ICR data, about 14,3% of the total number of variables were selected as discriminant signals based on their contributions in the covariance and in the correlation. Among these, close to 50% appear as uptrend with respect to CA.

**Figure 6:**
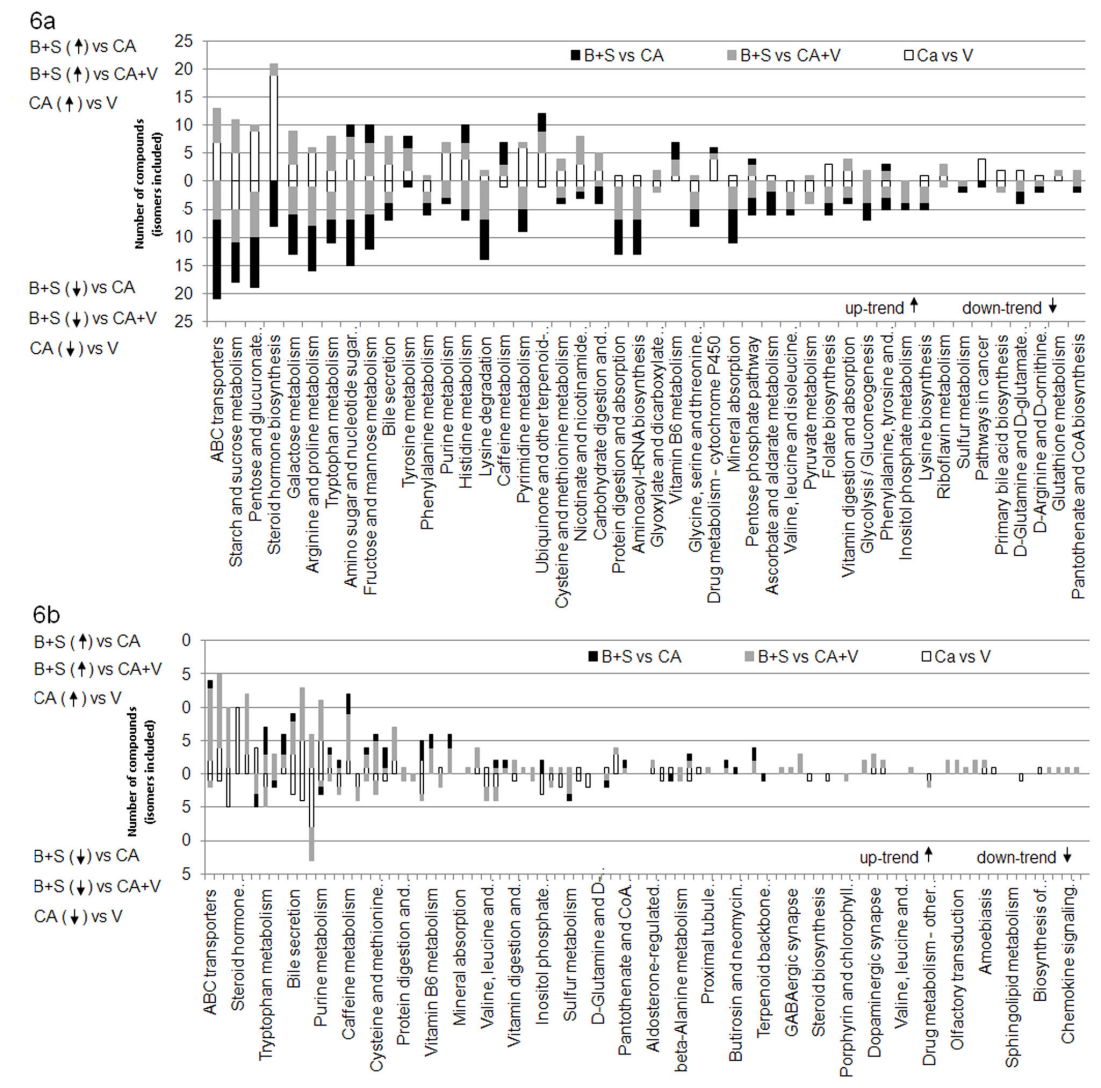
Metabolic pathways corresponding to discriminating signals (ESI+) annotated in MassTRIX (a). Metabolic pathways corresponding to discriminating signals (ESI-) annotated in MassTRIX (b).

The discriminant signals are loaded into MassTRIX and the metabolic pathways presenting the highest number of annotated compounds are shown in Figure 6a (white columns). A large part of the uptrend metabolites were identified as belonging to steroid hormone biosynthesis, pentose and glucuronate interconversions, starch and sucrose metabolism, ABC transporters, galactose metabolism and some amino acids metabolism (arginine, purine, hystidine and proline). The metabolites having downtrend relative intensities are situated on the starch and sucrose and pentose and glucuronate interconversions pathways.

In the negative mode, among the 11% of the discriminating signals, almost 4% have an uptrend behavior with respect to the group of CA. As in the positive mode the steroid hormone biosynthesis and pentose and glucuronate inetconversion pathways appear to have different trends in the two groups (Figure 6b, white columns). Phenylalanine metabolism seems to be another important pathway since as much as 8 compounds have a downtrend profile with respect to CA. Unlike the positive mode which counts 18% discriminant signals annotated by MassTRIX, the negative mode, which is less universal, counts around 9% annotated discriminant signals.

### (S vs CA) and (B vs CA): Salbutamol/Budesonide treated athletes vs clean athletes

As previously, the OPLS models were built for each of the two comparisons and for each of the two ionization modes. The comparison between the clean athletes and the salbutamol treated athletes (S vs CA) shows good performance indicators, irrespective of whether we look to the positive or to the negative mode. On the contrary, the model opposing the clean athletes and the budesonide treated athletes (B *vs* CA) is weaker and the p-value for the cross-validation is above the considered threshold (0.05) (see Table 1). This observation could be explained either by a weaker effect of medication or by an insufficient number of samples, as seen elsewhere.

**Figure 7:**
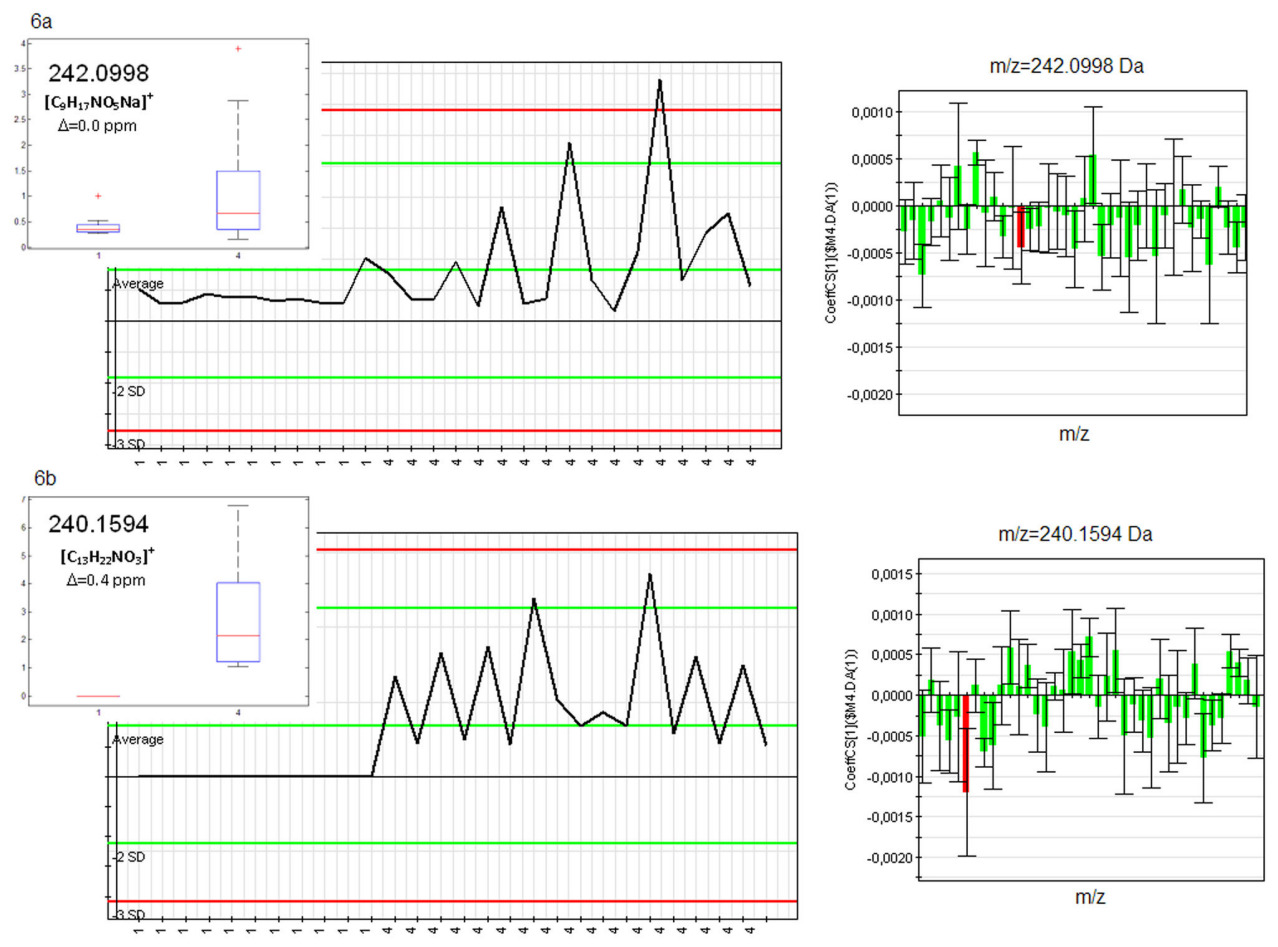
S vs. CA discriminant profiles: (a) pantothenic acid and (b) salbutamol.

Among the 1602 discriminant masses (~7%) selected in positive electrospray, salbutamol was found as a highly discriminant signal (Figure 7b). Its presence was confirmed by exact mass measurement (error 0.1ppm) and the assessment of the isotopic profile and testifies to the feasibility of this approach.

**Figure 8:**
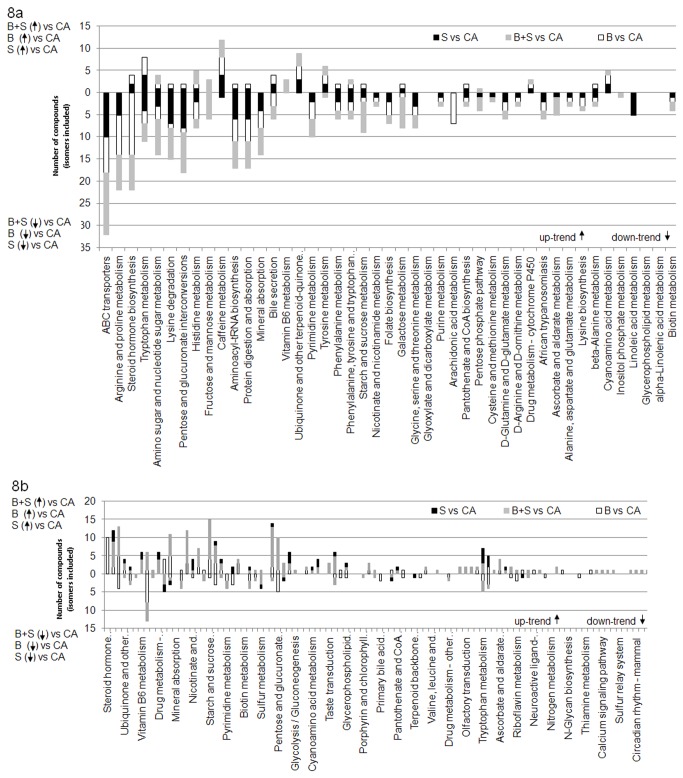
Metabolic pathways corresponding to discriminating signals (ESI+) annotated in MassTRIX (a). Metabolic pathways corresponding to discriminating signals (ESI-) annotated in MassTRIX (b).

Figure 8a shows a higher number of downtrend then uptrend signals with respect to salbutamol treated athletes (black columns). Also, the metabolic pathways that seem to be modified are those involving protein digestion, amino acids (arginine, proline, tryptophan, and lysine) [37-41] and pentose. According to the same figure, budesonide intake plays an important role in the biological reactions belonging to pathways such as steroid hormone biosynthesis. This could be explained by the fact that budesonide is a glucocorticoid itself.

In negative mode (Figure 8b) the trend is reversed and only a few uptrend variables are highlighted. The number of annotated compounds remains nevertheless lower than in positive mode and the majority of the compounds are issued from the model concerning the salbutamol treated athletes (~0.54% from the totality of examined variables).

### Similarities and differences between salbutamol and budesonide treated athletes

**Figure 9:**
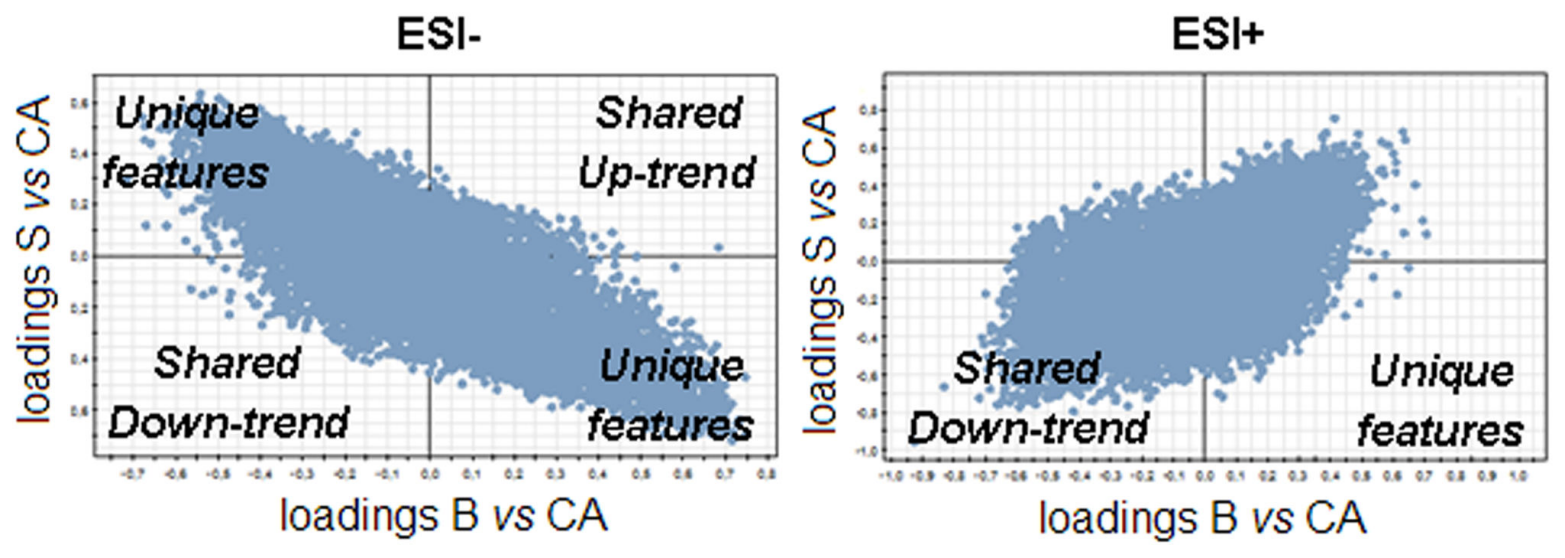
Up and downtrend features shared by the two models in the positive and negative mode.

SUS-plot is a graphical tool used to compare two statistical models (Figure 9). It indirectly finds the shared and the unique features of the groups that constitute the models. In brief, the SUS-plot is obtained by plotting the loading vector of a first model against the loading vector of the second model.

Pathways like ABC transporters appear as modified by both treatments to a similar degree. Steroid hormone biosynthesis pathway is modified mainly by budesonide and only in a small degree by salbutamol. In turn, pentose and glucose pathway is manly modified by salbutamol and to a smaller extent by budesonide. Surprisingly, fructose and mannose metabolisms do not appear to be modified when the two treatments are considered separately. In contrast, only when the two types of samples are considered as a single group (BS vs CA, Figure 1) they appear to be modified. Arachidonic acid and vitamin B6 metabolisms are modified by budesonide only, while linoleic acid metabolism or biosynthesis of unsaturated fatty acids are specific to salbutamol treatment (Figure 8a and 8b).

### Legitimacy of including volunteers in the experimental setup

As seen previously, a clear difference between the volunteers and the clean athletes can be made at metabolic level by using a global approach. In order to study the legitimacy to associate the volunteers to clean athletes, two models were compared in positive electrospray: (BS *vs* CA) and (BS *vs* CA+V).

As it can be seen in Figure 6a, the number of downtrend compounds annotated in MassTRIX is basically not influenced by the introduction of volunteers in the group of clean athletes (grey and black segments). However, for the uptrend metabolites, the metabolic differences between the athletes and the volunteers seem to have a greater impact on the comparison between treated (BS, budesonide and salbutamol together) and clean individuals (V+CA, athletes and volunteers together). This is, for example, the case of steroid hormone biosynthesis pathway. In figure 6a, we can see that when considering (BS *vs* CA) there are no uptrend annotations (black segment). However, a small segment of uptrend metabolites appears when associating the volunteers to the group of clean athletes (grey segment).

### Canonical Correlation Analysis for the comparison of two analytical techniques

As stated previously, the two analytical platforms were used independently to obtain the molecular fingerprints of each urine sample: UHPLC-QTOF/MS and FT-ICR/MS and the models resulted in similar performance indicators (Table 1). The latter has the advantage of having a very high resolution and 200 ppb precision, while the former one includes a possible separation step before the mass spectrometer which limits ionization suppression phenomena. By considering a tolerance of 5 mDa, we found that about 21% of the signals detected in FT-ICR are common to both data sets Thus, approximately 15% of the discriminating signals whose profiles were visually checked were detected in both data sets and almost 57% came from the FT-ICR data (see Table S1 for discriminating compounds as annotated by MassTRIX).

Canonical Correlation Analysis preceded by Principal Components Analysis (PCA-CCA) was used in order to compare the multivariate information contained in the two data sets. PCA was applied prior to the CCA in order to deal with the multicolinearity present in this type of data, to reduce the dimensionality, and thus, ease the interpretation of the results. As an example, this method was used for the two data sets corresponding to the model opposing the volunteers and the clean athletes (V vs CA) in negative mode. For each of the two data sets the 5 first principal components (loadings) were selected for further analysis. In each case, the principal components considered for CCA analysis accounted for about 50% of the total variance.

The analysis yielded five canonical functions explaining 96.6%, 71.5% 57.1%, 28.9% and 14.7% of the total variance shared by the two data sets. The full model, as well as the association of functions (f2,f3,f4,f5) and (f3,f4,f5) are considered statistically significant since the associated p-values are less than 0.05 (Table 2). On the contrary, the associations of function 4 and 5 and function 5 alone are not statistically significant and therefore not interpreted.

**Table 2:** Significance parameters for the Canonical Correlation Analysis.

	f1	f2	f3	f4	f5
squared canonical correlations coefficients	0.96	0.715	0.571	0.289	0.147
associated p-values	6.84e-07	0.008	0.043	0.144	0.109

**Figure 10:**
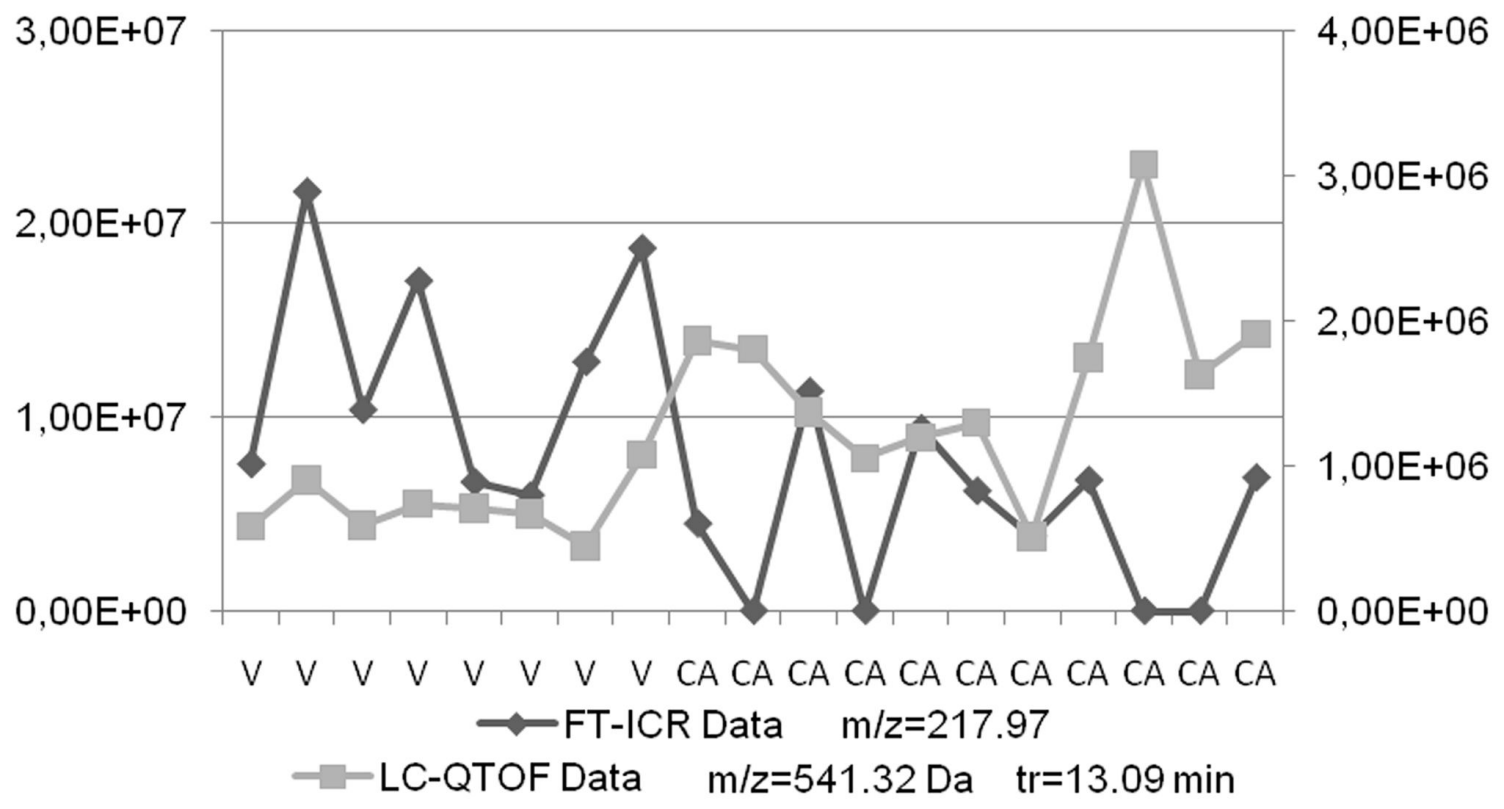
Negatively correlated variables emphasized by Canonical Correlation Analysis.

As for the OPLS models, the inspection of the canonical coefficients and structure coefficients reveal highly correlated principal components. Negatively or positively correlated m/z variables can be emphasized by considering these principle components (loadings). One such example is presented in Figure 10. The two correlated signals (m/z=217.97 and m/z=541.32) have down and up trend profiles, respectively. They prove to contribute greatly to the first principal components showing a clear discrimination between the two groups.

Thus, the CCA shows that, globally, the information contained in the LC-TOF data set is the same with the one contained in the FT-ICR data set.

## Conclusions

The non-targeted analysis of forty-six human urine samples was done on two analytical platforms: UHPLC-QTOF/MS and FT-ICR/MS. Given the different origins of the samples (V=Volunteers, CA=Clean Athletes, S=Salbutamol and B=Budesonide) several groups were created. These groups were firstly studied separately and then compared in order to emphasize possible similarities or differences between the samples. The statistical models created by using the OPLS method show good predictability for two models: CA *vs* V and S *vs* CA and lead to the selection of several discriminating variables. Significant advances towards annotation identification and interpretation have been made thanks to the high-resolution and high-precision measurements with the FT-ICR/MS and due to MassTRIX. More precisely, a significant number of the discriminated signals were annotated and placed on metabolic pathways.

The Canonical Correlation Analysis showed a strong correlation between the UHPLC-QTOF/MS data and the FT-ICR data.

The non-targeted metabolomics approach proved to be a helpful strategy even when real urine samples coming from a heterogeneous population were considered. In order to confirm the results presented in this paper and take into account the large inter-human variability, the same approach should be repeated on a larger cohort of individuals. Further studies are also needed in order to investigate the effect of other stimulants and doping substances.

## Supporting Information

Table S1 **List of compounds as annotated by MassTrix.**
(DOC)
