# Supplementary material for: Doping Control Using High and Ultra-High Resolution Mass Spectrometry Based Non-Targeted Metabolomics-A Case Study of Salbutamol and Budesonide Abuse
Source: PLoS One. 2013 Sep 18;8(9):e74584. doi: 10.1371/journal.pone.0074584 (PMC3776818; doi:10.1371/journal.pone.0074584)
Supplement: Table S1 — List of compounds as annotated by MassTrix. (DOC) [file pone.0074584.s001.doc]

| V vs CA | | | | | | |
| --- | --- | --- | --- | --- | --- | --- |
| **m/z** | **Form** | **Compound M** | **Group** | **Δppm** | **mSigma** | **Tr (min)** |
| FT-ICR & LC-qToF signals | | | | | | |
| 159.0278 | [M+Na]^+^ | C_5_H_4_N_4_O | CA | 0.28 | > 30 | 1.7 |
| 348.1354 | [M-H]^-^ | C_20_H_19_N_3_O_3_ | CA | 0.12 | <30 | 10.7 |
| 227.044  228.0473 | [M-H]^-^ | C_5_H_13_N_2_O_6_P | V | 0.77 | > 30 | 18.4 |
| 281.2486 | [M-H]^-^ | C_18_H_34_O_2_ | CA | 0.11 | <30 | 30.4 |
| 307.0647  308.0682 | [M+Na]^+^ | C_10_H_12_N_4_O_6_ | CA | 0.16 | <30 | 6.3 |
| 385.1987  363.21 | [M+Na]^+^  [M+H]^+^ | C_21_H_30_O_5_ | CA | 0.17 | > 30 | 13.8 |
| 535.218  536.222 | [M-H]^-^ | C_27_H_36_O_11_ | CA | 0.02 | <30 | 13.6 |
| FT-ICR signals | | | | | | |
| 154.0588 | [M+Na]^+^ | C_4_H_9_N_3_O_2_ | V | 0.39 | 30 |  |
| 211.1305 | [M+Na]^+^ | C_10_H_20_O_3_ | V | 0.07 | > 30 |  |
| 241.0597  243.064 | [M-H]^-^ | C_6_H_15_N_2_O_6_P | V | 0.8 | > 30 |  |
| 262.091 | [M+Na]^+^ | C_9_H_13_N_5_O_3_ | CA | 0.14 | > 30 |  |
|  |  |  |  |  |  |  |
| 269.1143 | [M-H]^-^ | C_12_H_18_N_2_O_5_ | CA | 0.05 | >30 |  |
| 275.0751 | [M+Na]^+^ | C_10_H_12_N_4_O_4_ | CA | 0.08 | > 30 |  |
|  |  |  |  |  |  |  |
| 277.1158  278.1192 | [M+Na]^+^ | C_12_H_18_N_2_O_4_ | CA | 0.23 | <30 |  |
|  |  |  |  |  |  |  |
|  |  |  |  |  |  |  |
| 279.0951 | [M+Na]^+^ | C_11_H_16_N_2_O_5_ | CA | 0.44 | > 30 |  |
| 289.1154 | [M-H]^-^ | C_10_H_18_N_4_O_6_ | CA | 0.04 | >30 |  |
| 296.0929 | [M-H]^-^ | C_17_H_15_NO_4_ | V | 0.55 | >30 |  |
| 310.2013  311.2046  332.1831  -333.1865 | [M+H]^+^  [M+Na]^+^ | C_17_H_27_NO_4_ | CA | 0.03 | <30 |  |
| 324.023 | [M-H]^-^ | C_11_H_11_N_5_O_3_S_2_ | CA | 0.31 | >30 |  |
| 334.1762 | [M+H]^+^ | C_17_H_23_N_3_O_4_ | V | 0 | > 30 |  |
| 340.1885  341.1917 | [M+Na]^+^ | C_19_H_27_NO_3_ | CA | 0.58 | <30 |  |
|  |  |  |  |  |  |  |
| 348.1571 (349.1604) | [M+Na]^+^ | C_20_H_23_NO_3_ | CA | 0.5 | <30 |  |
| 359.0479 | [M+Na]^+^ | C_15_H_10_F_6_O_2_ | V | 0.61 | > 30 |  |
| 383.1829 | [M+Na]^+^ | C_21_H_28_O_5_ | CA | 0.02 | >30 |  |
| 387.2144 | [M+Na]^+^ | C_21_H_32_O_5_ | CA | 0.12 | > 30 |  |
|  |  |  |  |  |  |  |
| 399.1279 | [M+Na]^+^ | C_17_H_20_N_4_O_6_ | CA | 0.43 | > 30 |  |
| 395.163 | [M-H]^-^ | C_10_H_28_N_4_O_12_  C_8_H_16_N_18_O_2_ | CA | <1 | > 30 |  |
| 401.1726 | [M+Na]^+^ | C_24_H_26_O_4_ | CA | 0.61 | > 30 |  |
| 401.1935  402.1967 | [M+Na]^+^ | C_21_H_30_O_6_ | CA | 0.16 | <30 |  |
|  |  |  |  |  |  |  |
|  |  |  |  |  |  |  |
| 403.1881 | [M+Na]^+^ | C_24_H_28_O_4_ | CA | 0.22 | > 30 |  |
| 407.138 | [M-H]^-^ | C_9_H_24_N_6_O_12_ | CA | <1 | > 30 |  |
| 409.344 | [M+Na]^+^ | C_27_H_46_O | CA | 0.22 | 23 |  |
|  |  |  |  |  |  |  |
|  |  |  |  |  |  |  |
|  |  |  |  |  |  |  |
| UHPLC-QTOF signals | | | | | | |
| 128.11 | [M+H]^+^ | C_7_H_13_NO | V | 9.9 | <30 | 10.3 |
| 245.19 | [M+H]^+^ | C_13_H_12_N_2_O_3_ | V | 1.5 | <30 | 9.4 |
| 269.08 | [M+H]+ | C_10_H_12_N_4_O_5_ | CA | 5.2 | <30 | 4.2 |
| 385.31 | [M+H]^+^ | C_26_H_40_O_2_ | V | 0.7 | <30 | 16.6 |
| 413.21 | [M+H]^+^ | C_23_H_28_N_2_O_5_  C_24_H_24_N_6_O | V | <3 | >30 | 15.3 |
| 430.29 | [M+H]^+^ | C_22_H_35_N_7_O_2_ | V | 0.8 | <30 | 18.6 |
| 561.35 | [M+H]^+^ | C_33_H_44_N_4_O_4_  C_32_H_48_O_8_  C_29_H_40_N_10_O_2_  C_34_H_40_N_8_ | V | <4 | <30 | 16.6 |
|  |  |  |  |  |  | 17.8 |
|  |  |  |  |  |  |  |
|  |  |  |  |  |  |  |
| 579.36 | [M+H]^+^ | C_29_H_42_N_10_O_3_  C_32_H_50_O_9_  C_33_H_46_N_4_O_5_  C_28_H_46_N_6_O_7_ | V | <4 | <30 | 16.6 |
| 615.37 | [M+H]^+^ | C_32_H_54_O_11_  C_29_H_46_N_10_O_5_  C_28_H_50_N_6_O_9_  C_30_H_42_N_14_O  C_25_H_42_N_16_O_3_  C_27_H_54_N_2_O_13_ | V | <4 | <30 | 16.6 |
| 835.39 | [M+4H]^4+^ | ~25 formules | V | <5 | >30 | 12.5 |
| S vs CA | | | | | | |
| **m/z** | **Forme** | **Compound M** | **Groupe** | **Δppm** | **mSigma** | **Tr (min)** |
| FT-ICR & LC-qToF signals | | | | | | |
| 144.1 | [M+H]^+^ | C7H13NO3 | S | 4.8 | 5.1 | 1.1 |
|  |  |  |  |  |  |  |
| 240.1594 | [M+H]^+^ | C_13_H_21_NO_3_ | S | 0.2 | 10.8 | 4.7 ; 7.4 |
| FT-ICR signals | | | | | | |
| 262.091 | [M+Na]^+^ | C_9_H_13_N_5_O_3_ | CA | 0.14 | >30 |  |
|  |  |  |  |  |  |  |
|  |  |  |  |  |  |  |
| 320.1161 | [M+H]^+^ | C_13_H_21_NO_6_S | S | 0.3 | >30 |  |
| 337.9916 |  |  | S |  |  |  |
| 342.0981 | [M+Na]^+^ | C_13_H_21_NO_6_S | S | 0.1 | 21 |  |
| 353.2181 | [M+H]^+^ | C_17_H_28_N_4_0_4_ | S | 0.2 | >30 |  |
| 358.0721 | [M+K]^+^ | C_13_H_21_NO_6_S | S | 0.4  0.1 | 7.6  15 |  |
| 402.1305 |  |  | S |  |  |  |
| 433.175 |  |  | S |  |  |  |
| UHPLC-QTOF signals | | | | | | |
| 107.81 |  |  | CA |  |  | 1 |
| 108.08 | [M+H]+ | C_7_H_9_N | CA | 7.5 | 3.9 | 1.1 |
| 122.1 | [M+H]+ | C_8_H_11_N | CA | 0.4 | 4.9 | 1.5 |
|  |  |  |  |  |  |  |
|  |  |  |  |  |  |  |
|  |  |  |  |  |  |  |
|  |  |  |  |  |  |  |
|  |  |  |  |  |  |  |
| 166.05 | [M+H]+ | C_8_H_7_NO_3_ | S | 1.7 | 13.6 | 4.4 |
|  |  |  |  |  |  |  |
|  |  |  |  |  |  |  |
|  |  |  |  |  |  |  |
|  |  |  |  |  |  |  |
| 213.14 | [M+H]+ | C_14_H_16_N_2_ | CA | 1.2 | 8 | 2.7 |
| 242.02 | [M+H]+ | C_6_H_11_NO_5_S_2_ | S | 1.6 | 6.5 | 1.3 |
| B vs CA | | | | | | |
| **m/z** | **Forme** | **Formule M** | **Groupe** | **Dppm** | **mSigma** | **Tr (min)** |
| FT-ICR & LC-qToF signals | | | | | | |
| 399.1781 | [M+Na]^+^ [M+H]^+^ | C_21_H_28_O_6_ | B | 0.1 | > 30 | 12.8 |
|  |  |  |  |  |  |  |
|  |  |  |  |  |  |  |
|  |  |  |  |  |  |  |
|  |  |  |  |  |  |  |
| 401.1935 | [M+Na]^+^ [M+H]^+^ | C_21_H_30_O_6_ | CA | 0.06 | <30 | 10.3 et 12.5 |
|  |  |  |  |  |  |  |
|  |  |  |  |  |  |  |
|  |  |  |  |  |  |  |
|  |  |  |  |  |  |  |
|  |  |  |  |  |  |  |
|  |  |  |  |  |  |  |
| FT-ICR signals | | | | | | |
| 447.245 | [M+Na]^+^  [M+H]^+^ | C_22_H_36_N_2_O_6_  C_20_H_30_N_8_O_4_ | CA | 0.13  0.49 | < 30  <30 |  |
| 445.231 | [M+Na]^+^  [M+H]^+^ | C_22_H_34_N_2_O_6_  C_20_H_28_N_8_O_4_ | CA | 0.2  0.8 | > 30  > 30 |  |
| 383.1830 | [M+Na]^+^ | C_21_H_28_O_5_ | CA | 0.18 | > 30 |  |
|  |  |  |  |  |  |  |
|  |  |  |  |  |  |  |
|  |  |  |  |  |  |  |
|  |  |  |  |  |  |  |
|  |  |  |  |  |  |  |
| 385.1987 | [M+Na]^+^ | C_21_H_30_O_5_ | CA | 0.37 | > 30 |  |
|  |  |  |  |  |  |  |
|  |  |  |  |  |  |  |
|  |  |  |  |  |  |  |
|  |  |  |  |  |  |  |
|  |  |  |  |  |  |  |
|  |  |  |  |  |  |  |
| 387.2144 | [M+Na]^+^ | C_21_H_32_O_5_ | CA | 0.07 | >30 |  |
|  |  |  |  |  |  |  |
|  |  |  |  |  |  |  |
|  |  |  |  |  |  |  |
|  |  |  |  |  |  |  |
|  |  |  |  |  |  |  |
|  |  |  |  |  |  |  |
| 389.2299 | [M+Na]^+^ | C_21_H_34_O_5_ | CA | 0.3 | >30 |  |
|  |  |  |  |  |  |  |
|  |  |  |  |  |  |  |
|  |  |  |  |  |  |  |
|  |  |  |  |  |  |  |
| 251.1002 | [M+Na]^+^ | C_10_H_16_N_2_O_4_ | CA | 0.3 | 10.3 |  |
|  |  |  |  |  |  |  |
| 262.091 | [M+Na]^+^ | C_9_H_13_N_5_O_3_ | CA | 0.13 | >30 |  |
|  |  |  |  |  |  |  |
|  |  |  |  |  |  |  |
| UHPLC-QTOF signals | | | | | | |
| 433.2568 | [M+H]^+^ | C_25_H_36_O_6_  C_22_H_28_N_10_  C_21_H_32_N_6_O_4_ | - | 3.8  1.2  1.9 | 5.2  15  22 | 16.8 |
| 447.2372 | [M+H]^+^ | C_25_H_34_O_7_  C_26_H_30_N_4_O_3_  C_22_H_26_N_10_O  C_21_H_30_N_6_O_5_ | - | 1.2 | >30 | 14.7 |
| 118.08 | [M+H]^+^ | C_5_H_11_NO_2_ | CA | 5 | 2.6 | 1 |
|  |  |  |  |  |  |  |
|  |  |  |  |  |  |  |
|  |  |  |  |  |  |  |
|  |  |  |  |  |  |  |
|  |  |  |  |  |  |  |
|  |  |  |  |  |  |  |
|  |  |  |  |  |  |  |
| 279.13 | [M+H]^+^ | C_19_H_18_O_2_ | CA | 0.4 | 18.7 | 6 |
